# Pregabalin use in forensic hospitals and prisons in German speaking countries—a survey study of physicians

**DOI:** 10.3389/fpubh.2023.1309654

**Published:** 2024-01-08

**Authors:** Michal Novotny, Jan Bulla, Daniela Hubl, Sebastian Karl Maximilian Fischer, Martin Grosshans, Andreas Gutzeit, Oliver Bilke-Hentsch, Erich Seifritz, Jochen Mutschler

**Affiliations:** ^1^Private Clinic Meiringen, Willigen, Switzerland; ^2^Faculty of Medicine, University of Zurich, Zurich, Switzerland; ^3^Clinic of Forensic Psychiatry and Psychotherapy, Reichenau Centre of Psychiatry, Reichenau, Germany; ^4^Center of Forensic Psychiatry and Psychology, Universitäre Psychiatrische Dienste Bern (UPD), Bern, Switzerland; ^5^Psychiatric Services Lucerne, Lucerne, Switzerland; ^6^Department of Global Health, Safety and Well-Being, Systemanalyse Programmentwicklung Societas Europaea (SAP SE), Walldorf, Germany; ^7^Department of Radiology, Paracelsus Medical University, Salzburg, Austria; ^8^Department of Health Sciences and Medicine, University of Lucerne, Lucerne, Switzerland; ^9^Institute of Radiology and Nuclear Medicine and Breast Center St. Anna, Hirslanden Klinik St. Anna, Lucerne, Switzerland; ^10^Department of Psychiatry, Psychotherapy and Psychosomatics, Psychiatric Hospital, University of Zurich, Zurich, Switzerland

**Keywords:** pregabalin, misuse, prison, forensic, pharmacology, German-speaking countries, Switzerland, gabapentinoids

## Abstract

**Background:**

Pregabalin is a gamma-aminobutyric acid (GABA) analog that was approved in the EU in 2004 for the treatment of neuropathic pain, generalized anxiety disorder and epilepsy. Since its introduction, pregabalin abuse and misuse has increased significantly. In Switzerland, clinical reports suggest that pregabalin misuse is common among patients in forensic hospitals and prisons. However, data on pregabalin use is scarce, especially in these settings. Therefore, we conducted a study to explore patterns of pregabalin use among prison and forensic patients.

**Methods:**

We used a questionnaire to survey physicians working in prison and forensic medicine in German-speaking countries. A total of 131 responses were received.

**Results:**

According to the physicians' subjective assessment, 82.5% of them had observed a recent increase in pregabalin use by their patients and 89.1% of them reported that their patients requested pregabalin without a clear medical indication. Patients misusing pregabalin in combination with other illicit substances were observed by 93.3% of the physicians surveyed. According to 73.5% of the physicians surveyed, they had already encountered patients on pregabalin doses of more than 600 mg/day (the maximum recommended daily dose); the highest dose reported was 4,200 mg/day. According to 85.0% of physicians surveyed, they have observed patients experiencing withdrawal symptoms from pregabalin, with the most commonly reported symptoms being displeasure and high aggression. Regarding the nationality of pregabalin-misusing patients, 58.3% of the interviewed physicians reported to be rather in contact with foreign patients, mainly from Northwest Africa (Maghreb). Only 45.0% of the surveyed physicians prescribe pregabalin. Among patients who developed behavioral problems while taking pregabalin, none of the physicians (0.0%) showed a tendency to continue pregabalin at the same dose; all respondents chose to reduce/substitute/discontinue.

**Conclusion:**

Our study has provided confirmatory evidence that the use of pregabalin presents a significant issue in forensic and prison medicine across German-speaking countries. Prescribing pregabalin in this field can compound use disorder problems and exacerbate challenges in daily life for those in forensic institutions or prisons. It is necessary that all physicians who prescribe pregabalin are clearly informed about the management (including the risks) of this drug.

## 1 Introduction

Pregabalin is a gamma-aminobutyric acid (GABA) analog and, together with gabapentin, it belongs to the group of gabapentinoids (1). Pregabalin was introduced to the EU market in 2004 (2) and is approved in Europe for the treatment of neuropathic pain, generalized anxiety disorder and epilepsy (1). Gabapentinoids were initially created as GABA mimetics; however, they bind to the alpha-2-delta subunit of voltage-gated calcium channels and not the GABA receptors directly (3).

Since its introduction, there has been a rise in pregabalin abuse (4, 5). A database analysis conducted by the Poison Control Center in Munich revealed a rise in substance misuse of 2,267% from 2008 to 2015 (2). A recent study from Ireland showed an increase in deaths associated with pregabalin, while pregabalin was included in 240 (16%) toxicology reports of 1,489 poisoning deaths, with a significant increase from 15 (4.5%) in 2013 to 94 (26%) in 2016 (6). A significant increase in recreational use of pregabalin among French adolescents has been reported between 2004 and 2020, with 81% of the adolescents either being homeless or living in migrant shelters (7). Outside of Europe, the problematic use of pregabalin is also challenging. An Australian study presented that one in every seven individuals taking pregabalin appeared to be at high risk of misuse (8). A study from the US also found that more than 600 adverse drug events (ADEs) of gabapentinoid abuse were reported between 2012 and 2016, out of a total of more than 10,000 all-cause ADEs reported to the Food and Drug Administration Adverse Event Reporting System (9). Another study from the US by Evoy et al. (10) in 2021 showed that of 1,843 respondents, 121 (6.6%) reported a history of gabapentinoid misuse/abuse/non-prescription use. These results provided one of the first estimates in a nationally distributed sample of the general population in the United States.

The abuse potential of both gabapentinoids has been compared in several studies, which have found pregabalin to have a greater potential for abuse than gabapentin (11). Pregabalin has high bioavailability (>90%) regardless of dose, significantly faster absorption and six times greater binding affinity for the alpha-2-delta subunit compared to gabapentin (12). A Japanese study conducted in 2021 revealed various psychopathological withdrawal symptoms, including restlessness, anxiety, and insomnia. Additionally, vegetative withdrawal symptoms, such as sweating, heart palpitations and lacrimation, were described. The somatic withdrawal symptoms have included breathlessness, decreased appetite and headache. Other withdrawal symptoms may include hallucinations, suicidal thoughts and tremor (13).

A systemic review update in 2021 found evidence that misuse and abuse of gabapentinoids is an increasing trend and causes significant harm to patients. Accordingly, health professionals should exercise caution when prescribing pregabalin to high-risk individuals and monitor them for signs of misuse or abuse (14). There has been a significant increase in pregabalin sales in Switzerland, with pharmacy sales of pregabalin increasing between 2015 and 2022. Over this time frame, pharmacy sales escalated by over a third (36.9%), with 524,498 packs sold in 2022 as opposed to 383,256 packs sold in 2015 (15).

However, a study conducted by Mutschler et al. on the misuse of pregabalin by opioid-dependent individuals in Switzerland found no traces of the drug in hair samples. These findings are in sharp contrast to reports of misuse of pregabalin by opioid-dependant patients in other countries (16). It seems that a country's drug policy has an impact on the illicit use of pregabalin (2).

Available data suggested that abuse of pregabalin is more common in certain countries or in certain populations (2). In Switzerland, clinical reports suggest that patients misusing pregabalin are often found in forensic hospitals and prisons (according to personal physician reports). This presents a challenge in managing patients with severe withdrawal symptoms and behavioral problems. As the data on pregabalin misuse especially in prisons and forensics is very limited, we have designed a study to generate more knowledge about misuse of pregabalin in this field of medicine. Additionally, the purpose of this study is to enhance clinical knowledge and offer improved therapeutic support to a severely affected patient group. We anticipate that our study will enhance the understanding of problematic use of pregabalin.

## 2 Materials and methods

### 2.1 Study design

The questionnaire used for our anonymous survey was developed and coordinated with medical experts in forensics and prison medicine from Germany and Switzerland. The survey was designed und distributed in two ways.

First, the questionnaire was created using the online survey tool “www.onlineumfragen.com” and distributed online. Participants from Switzerland were contacted via the mail distributor of the Swiss Society of Forensic Psychiatry (SSFP) and via the co-authors of this study. Participants from Germany and Austria were contacted via the mail distributor of the co-author of our study. In addition, the other various forensic and prison institutions were contacted, in which case the contact details of other institutions were obtained from internet websites. Finding participants for our study on the internet was made more difficult by the lack of transparency of prison and forensic websites—physicians are usually not officially mentioned due to their protection. Out of all the German federal states, 35 institutions were contacted via info mail from the home page of their websites. Participants in our study were also asked to forward the link of our questionnaire among other colleagues.

Secondly, the questionnaire was distributed on site at the 7th Prison Medicine Days (7. Gefängnis Medizin-Tage) in Frankfurt am Main, Germany.

The survey questionnaire contained a total of 30 questions, covering the following topics: characterization of patients, subjective assessment of physicians, procurement of pregabalin, prescription patterns of physicians and therapy/management of problems. Participants were also asked about eight different biographical questions (Table 1). The response option “I don't know/no answer” could also be selected. The questionnaire did not clearly define the terms “abuse” and “misuse.” Therefore, the more inclusive term “misuse” was used throughout this study.

**Table 1:** Demographic characteristics of the 131 participating physicians.

**Characteristic**	***n* (%)**
**Gender**	123 (100.0)
Female	64 (52.0)
Male	59 (48.0)
**Country of practice**	125 (100.0)
Germany	92 (73.6)
Switzerland	27 (21.6)
Austria	5 (4.0)
Luxembourg	1 (0.8)
**Institution where practicing**	124 (100.0)
Judicial environment	65 (52.4)
Medical environment (forensic clinics)	59 (47.6)
**Specialist certificate**	125 (100.0)
With specialist certificate	95 (76.0)
Without specialist certificate	30 (24.0)

### 2.2 Sample

A link to the online survey was sent by e-mail to various forensic institutions and prisons in Germany, Switzerland and Austria. The contact details of these institutions were acquired from publicly accessible sources, e.g., internet websites. Secondly, the questionnaire was distributed on site at the event “7th Prison Medicine Days” in Frankfurt am Main in Germany, which took place from 01.12.2022 to 02.12.2022.

### 2.3 Statistical procedures

The descriptive presentation of continuous variables was done as mean, partly in combination with the standard deviation. For categorical variables were given the absolute and relative frequencies.

An inquiry to the Ethics Committee of Zurich revealed that no ethics approval is required for this study.

## 3 Results

The survey was only set up to physician working in forensic facilities or in prisons. Out of 165 people, 73 (44%) completed the online survey. A total of 58 responses were collected from the 7th Prison Medicine Days event, out of a total of ~200 participants (29%). A total of 131 responses have been collected. Participants were on average 50.6 years old (SD = 11.48) and had an average of 23.4 years of clinical experience (SD = 10.95).

### 3.1 Characterization of patients

A total of 115 (89.1%) interviewed physician respondents reported to observe patients requesting pregabalin even if there is no clear medical indication. In addition, patients misusing pregabalin in combination with other illicit substances were observed by 93.3% (*n* = 112) of the physicians surveyed. Overall, 83 (73.5%) of the physicians surveyed had already encountered patients on pregabalin doses higher than 600 mg/day (maximum recommended daily dose); the highest dose reported in our survey was 4,200 mg/day. A total of 66 (71.0%) interviewed physicians responded that their patients had been taking pregabalin for < 5 years.

**Figure 1:**
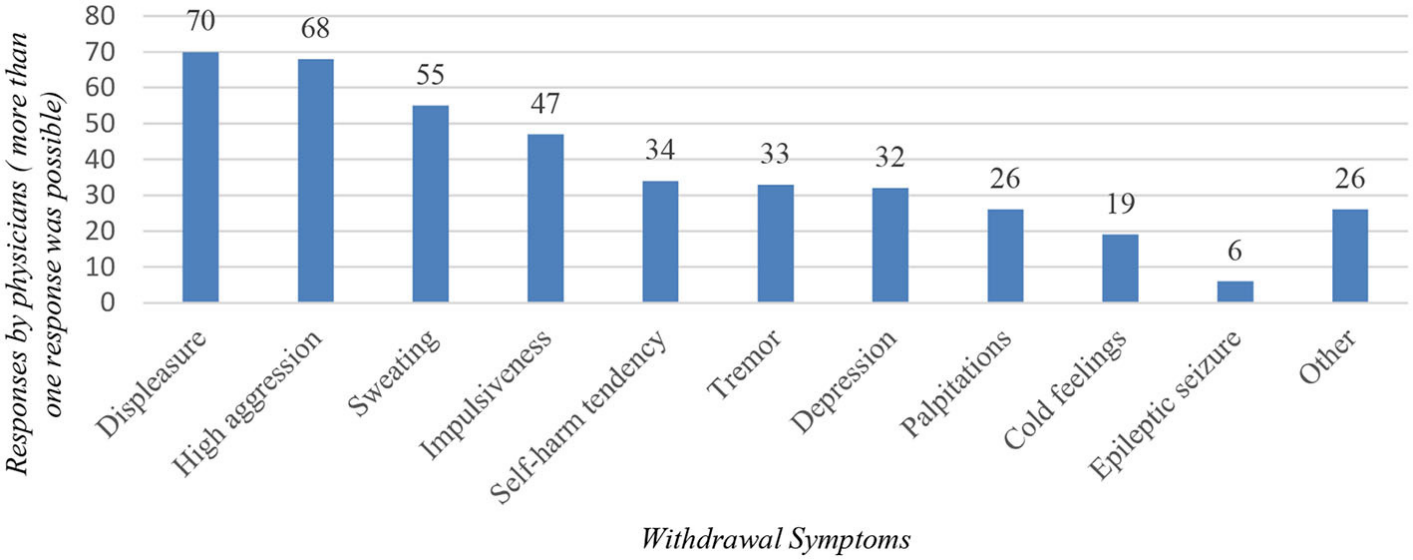
Withdrawal symptoms from pregabalin observed by interviewed physicians.

A total of 102 (85.0%) interviewed physicians reported that they have observed patients experiencing withdrawal symptoms from pregabalin, in which case the most frequently mentioned symptoms were displeasure and high aggression (Figure 1). An epileptic seizure as a withdrawal complication was already observed by 5.4% (*n* = 6) of physicians surveyed. Patients experiencing behavioral problems were observed by 61.4% (*n* = 70) of respondents, of which “external aggression” was selected by 51 respondents, “self-aggression” was selected by 17 respondents and two respondents selected the option “other.”

**Figure 2:**
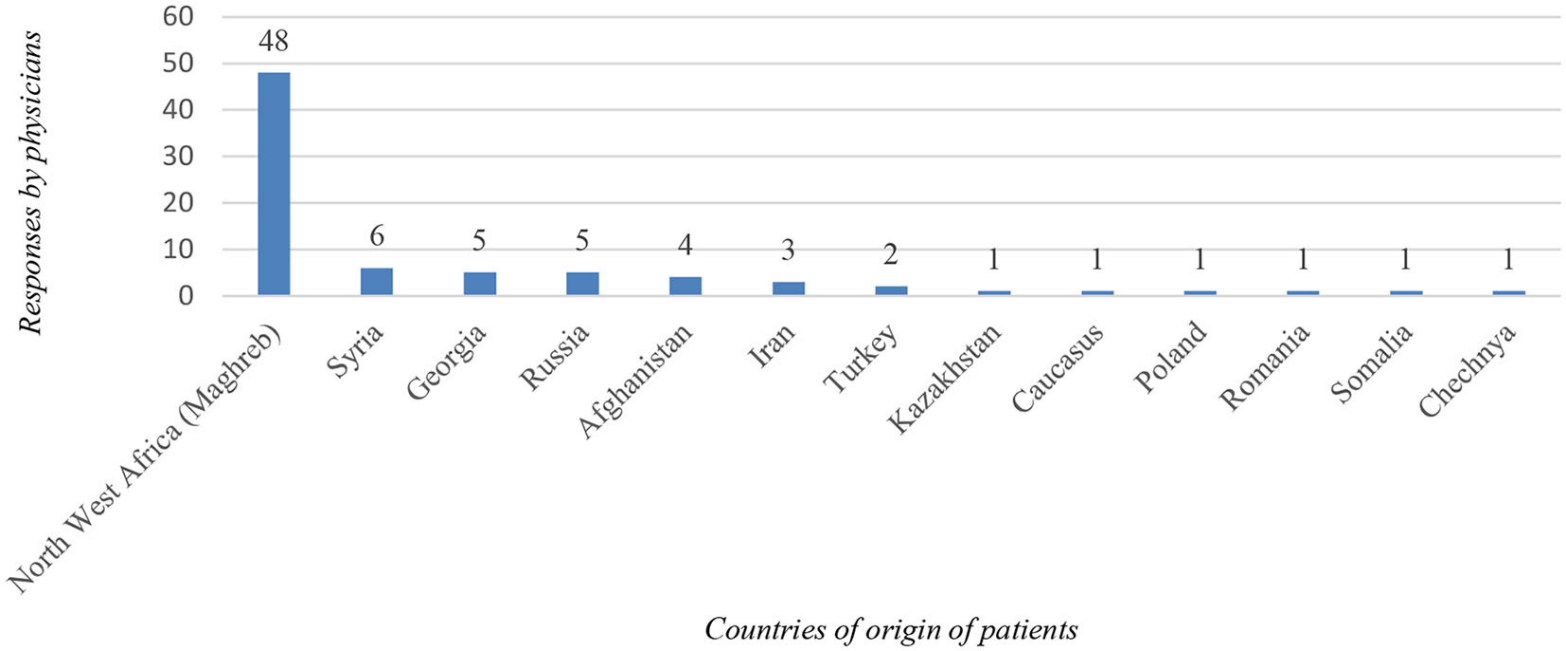
Illustration of the countries from which, according to the interviewed physicians, most of the foreign patients originate.

Regarding the nationality of pregabalin-misusing patients, 58.3% (*n* = 56) of the interviewed physicians reported to be rather in contact with foreign patients. Respondents identified many countries of origin of foreign patients misusing pregabalin, with Northwest Africa (Maghreb) being the most common (*n* = 48; Figure 2). When asked whether patients bring pregabalin with them from their home country, only 41.0% (*n* = 34) of physicians answered in the affirmative.

### 3.2 Subjective assessment of physicians, procurement of pregabalin

According to the subjective assessment of the physicians surveyed, 82.5% (*n* = 101) of them have recently observed an increase in the use of pregabalin by their patients. A total of 52 (62.7%) of the physicians surveyed reported being in contact with patients whose use of pregabalin was initiated on the illicit market, while 31 (37.3%) of the respondents reported being in contact with patients whose use of pregabalin was initiated iatrogenically. Regarding the source of pregabalin, 71.4% (*n* = 90) of interviewed physicians observed patients obtaining pregabalin from the illicit market as well as from physicians prescription, whereas 19.8% (*n* = 25) of the physicians surveyed observed the patients obtaining pregabalin only from illicit market and 8.7% (*n* = 11) of physicians observed patients becoming pregabalin only from a physician prescription.

### 3.3 Diagnostics

With regard to diagnosis, physicians were asked about the use of drug screening. According to 63.3% (*n* = 81) of respondents, drug screening is routinely carried out on admission. When drug screening is performed, it is both qualitative and quantitative in 51.3% (*n* = 41) of cases. Only 37.6% (*n* = 47) of the respondents test their patients for the presence of pregabalin.

### 3.4 Prescription patterns of physicians

Only 45.0% (*n* = 58) of respondents prescribe pregabalin themselves for the treatment of peripheral and central neuropathic pain/epilepsy/generalized anxiety disorder. Only 9.6% (*n* = 12) of respondents prescribe pregabalin OFF-label for the treatment of withdrawal symptoms associated with opioid dependence. Of all respondents to this question (*n* = 123), only colleagues from Germany (*n* = 11) and Austria (*n* = 1) answered positively, while all Swiss colleagues answered negatively.

### 3.5 Therapy/management of problems

Among patients who developed behavioral problems while taking pregabalin, no physician (0.0%) showed a tendency to continue pregabalin at the same dose. All responders (*n* = 73) opted for reduction/substitution/discontinuation. The medications mentioned for substitution were: benzodiazepines (*n* = 7), gabapentin (*n* = 7), valproic acid (*n* = 3) and sedating neuroleptics (*n* = 2). For doses above 600 mg/day, 75.2% (*n* = 85) of respondents chose to reduce, 13.3% (*n* = 15) to stop, 8.8% (*n* = 10) to substitute and only 2.7% (*n* = 3) to continue. The most common substitution option by doses above 600 mg/day was the drug gabapentin (*n* = 5).

## 4 Discussion

The objective of this research was to evaluate the experiences of forensic and prison physicians in German-speaking countries with the use of pregabalin and the recently reported problems associated with its increasing abuse and misuse. Improved knowledge of the clinical management of patients with problematic use of pregabalin is urgently needed, both to improve patient care and to increase theoretical knowledge of the problem.

Hence, this study undertook a systematic survey of physicians specializing in forensic or prison medicine. Several topics were addressed, the most important of which was the characterization of the patients taking pregabalin that these physicians were treating. Furthermore, the topics of misuse and behavioral problems while taking pregabalin were inquired. In addition, the subjective assessment and prescribing patterns of physicians have been assessed. Finally, the study looked at the diagnostics, therapy and management of the whole issue.

The findings demonstrate that administering pregabalin to prison and forensic patients is a major challenge for the physicians who took part in our study. Patients misusing pregabalin in combination with other illicit substances were observed by majority of physicians surveyed. The concomitant use of other drugs while misusing pregabalin has also been described in various studies (14). The physicians surveyed reported a perceived rise in pregabalin consumption among their patients. Pharmacy sales of pregabalin in Switzerland have also increased by over a third between 2015 and 2022 (15).

Another aspect to consider is the occurrence of withdrawal symptoms. Our research revealed that participants frequently reported experiencing increased feelings of displeasure and heightened levels of aggression as common withdrawal symptoms among their patients. These manifestations can be particularly detrimental to forensic and prison medicine. Patients who experienced behavioral problems while taking pregabalin were not more likely to be continued on pregabalin at the same dose, with all physicians opting to reduce, substitute or discontinue the drug. A case report from 2018 (17) documented a patient who suffered from withdrawal symptoms for a few weeks, while other case series (13) indicate that withdrawal symptoms may arise even at usual doses and during short-term use of pregabalin, emphasizing the significance of slow and vigilant reduction of pregabalin with careful monitoring of withdrawal symptoms from this drug. The physicians in our study most commonly substituted pregabalin with one of following medications: benzodiazepines, gabapantine, and valproic acid.

The majority of physicians surveyed reported encountering foreign patients who had problems with pregabalin, with most respondents reporting observing patients from Northwest Africa (Maghreb). Another study from Switzerland also highlighted the problem of pregabalin abuse among Maghreb patients in Swiss prisons, where aggressive behavior was often described in order to obtain a prescription for pregabalin (18). The reason why patients from Northwest Africa seem more prone to pregabalin-related problems remains unclear. More research should be done in these countries, looking at the illicit market and physicians' prescribing patterns. The prevention of pregabalin misuse in these countries should also be more rigorous. Another study noted that prescription medications like pregabalin and clonazepam have lately become increasingly accessible and affordable in MENA (Middle East and North Africa) countries, and according to Portela et al., the misuse of these substances in Europe is expected to increase due to migration flows from this region (19).

Less than half of the respondents reported that they perform drug screening for pregabalin. It should be strongly indicated in these patients. Most importantly, physicians should be very cautious about prescribing pregabalin to patients with a history of substance-related disorders (20, 21). Less than half of the physicians surveyed prescribe pregabalin for the approved indications of neuropathic pain, generalized anxiety disorder and epilepsy. This is an indication that prescribers are reluctant to use pregabalin as a result of negative experiences. Pregabalin is used for the treatment of withdrawal symptoms in opioid dependence by only a tenth of respondents, while no physician from Switzerland prescribed pregabalin for this OFF-label indication, which may reflect the more liberal policy on the use of opioid substitution in Switzerland (2). It is crucial to emphasize that individuals who abuse opioids represent a high-risk category for the misuse of gabapentinoids (22).

The most recent study from Ireland looked at trends in the prescribing of opioids, benzodiazepines, Z-drugs and gabapentinoids in Irish prisons between 2012 and 2020. In this case, opioid prescribing rates were cut in half between 2015 and 2020, and benzodiazepine and Z-drugs prescribing rates followed a downward trend. In contrast, gabapentinoid prescribing rates increased over the study period (although they appear to be decreasing in 2020), which was largely driven by pregabalin (23). It has been reported that there has been an overall decrease in gabapentinoid prescribing in prisons in New South Wales in recent years (24), suggesting that this issue is also present in Australia.

Our study has shown that the use of pregabalin in forensic and prison medicine in German-speaking countries is a significant challenge. An additional prescription of pregabalin in this area of medicine can lead to a new, additional use disorder problem, which can make everyday life in a forensic institution or prison much more difficult. It is important to note that individuals in prison or forensic settings are at particular risk for developing pregabalin use disorder, as are individuals with a history of substance use disorder, and that the two populations often overlap significantly (12, 18). It is essential that all physicians who prescribe pregabalin are clearly informed about the management (including the risks) of this drug. Another issue that arises from the fact that abuse of pregabalin has been described in several publications is whether certain prescription restrictions should be implemented. Prescribing gabapentinoids requires very careful and individualized benefit:risk stratification, along with monitoring for efficacy, harms, and adherence, and vigilant tapering/discontinuation when risks begin to outweigh benefits (25), as is the case with prescribing opioid analgesics, medical cannabis, benzodiazepines, and other prescription drugs suitable for misuse. However, it is very important to point out that gabapentinoids are a very useful and relatively safe pharmacological tool not only for the treatment of neuropathic pain and some neuropsychiatric disorders, but also in the world of addiction medicine (25, 26).

## 5 Limitations

The questionnaire was only administered to physicians working in prisons or forensic hospitals in German-speaking countries. This sample therefore does not cover other European countries and especially not the countries of the Northwest African region (Maghreb). Potential future studies should include physicians from these countries. Furthermore, our sample size was relatively small with only 131 physicians. There may have been bias in provider selection through personal emails with study authors and it cannot be ruled out that only a certain group of physicians responded to the survey. In addition, specific local treatment pathways within prisons regarding the prescription of medications could not be captured in this study. Furthermore, the results may have been influenced by recall bias on the part of the physicians surveyed. Nevertheless, our study is one of the first to provide insight into the misuse of pregabalin in specialized healthcare settings (forensic/prison) in German-speaking countries and therefore enables a better clinical management of these clinically challenging patients.

## 6 Conclusion

Our study has provided confirmatory evidence that the use of pregabalin presents a significant issue in forensic and prison medicine across German-speaking countries. Prescribing pregabalin in this field can compound use disorder problems and exacerbate challenges in daily life for those in forensic institutions or prisons. It is necessary that all physicians who prescribe pregabalin are clearly informed about the management (including the risks) of this drug.

## Data availability statement

The original contributions presented in the study are included in the article/Supplementary material, further inquiries can be directed to the corresponding author.

## Ethics statement

Ethical review and approval was not required for the study on human participants in accordance with the local legislation and institutional requirements. Written informed consent from the participants was not required to participate in this study in accordance with the national legislation and the institutional requirements.

## Author contributions

MN: Conceptualization, Data curation, Project administration, Formal analysis, Writing – original draft, Writing – review & editing. JB: Writing – review & editing. DH: Writing – review & editing. SF: Writing – review & editing. MG: Writing – review & editing. AG: Writing – review & editing. OB-H: Writing – review & editing. ES: Writing – review & editing. JM: Conceptualization, Data curation, Project administration, Formal analysis, Supervision, Writing – review & editing.

## 7 Questionnaire

Questions for physicians in the field of forensics/prison medicine regarding the increasing use of Pregabalin [Fragen an Ärzte im Bereich Forensik/Gefängnismedizin bzgl. des zunehmenden Konsums von Pregabalin] – see Supplementary material.
