# Supplementary material for: Pregabalin use in forensic hospitals and prisons in German speaking countries—a survey study of physicians
Source: Front Public Health. 2024 Jan 8;11:1309654. doi: 10.3389/fpubh.2023.1309654 (PMC10800468; doi:10.3389/fpubh.2023.1309654)
Supplement: Supplementary file 1 [file Data_Sheet_1.PDF]

Fragen an Ärzte im Bereich Forensik/Gefängnismedizin bzgl. des zunehmenden Konsums von Pregabalin (Lyrica®)

Sehr geehrte Kolleginnen und Kollegen,

gerne möchten wir Sie herzlich zur Befragung bezüglich des Konsums von Pregabalin (Lyrica®) im Bereich Forensik/Gefängnismedizin einladen. Der Missbrauch von Pregabalin (Lyrica®) nahm seit der Markteinführung im 2004 zu, ebenso die Zahl der Publikationen hierzu.

In letzter Zeit wurde besonders im Bereich Forensik/Gefängnismedizin in deutschsprachigen Ländern eine Problematik im Umgang mit Pregabalin (Lyrica®) beobachtet, hier ist die Datenlage jedoch noch ungenügend. Durch unsere Umfrage erhoffen wir uns, dass in einem ersten Schritt die Erkenntnis zum Missbrauch von Pregabalin (Lyrica®) verbessert wird.

Gerne laden wir Sie daher ein, den beiliegenden Fragebogen auszufüllen. Der Aufwand beträgt max. 10 Minuten.

Herzlichen Dank, dass Sie bei dieser Umfrage mitmachen!

Freundliche Grüsse

Prof. Dr. med. Jochen Mutschler, Chefarzt, Privatklinik Meiringen
Michal Novotny, Assistenzarzt, Privatklinik Meiringen

Fragen an Ärzte im Bereich Forensik/Gefängnismedizin bzgl. des zunehmenden Konsums von Pregabalin (Lyrica®)

1) <u>Wird Ihren Patienten/Patientinnen Pregabalin (Lyrica®) ärztlich verordnet oder erhalten sie es vom Schwarzmarkt?</u>
   a) *Vom Arzt verschrieben*
   b) *Schwarzmarkt*
   c) *Beides*

2) <u>Fordern Ihre Patienten/Patientinnen die Verordnung von Pregabalin (Lyrica®) ein, auch wenn keine klare ärztliche Indikation besteht?</u>
   a) *Ja*
   b) *Nein*

3) <u>Führen Sie bei allen Patienten/Patientinnen in der Aufnahmesituationen ein Drogenscreening durch?</u>
   a) *Ja*
   b) *Nein*

4) <u>Falls ja, wie führen sie das Drogenscreening durch?</u>
   a) *Qualitative*
   b) *Quantitative*
   c) *Qualitativ und Quantitativ*

5) <u>Testen Sie Ihre Patienten/Patientinnen auf das Vorhandensein von Pregabalin (Lyrica®)?</u>
   a) *Ja*
   b) *Nein*

6) <u>Verordnen Sie selber bei Ihren Patienten/Patientinnen Pregabalin (Lyrica®) zur Behandlung peripherer und zentraler neuropathischer Schmerzen/Epilepsie/generalisierten Angststörungen?</u>
   a) *Ja*
   b) *Nein*

7) <u>Sind Ihre Patienten/Patientinnen mit Pregabalin (Lyrica®) zusätzlich von anderen illegalen Substanzen abhängig?</u>
   a) *Ja*
   b) *Nein*

8) <u>Merken Sie eine Zunahme im Konsumverhalten von Pregabalin (Lyrica®) bei Ihren Patienten/Patientinnen?</u>
   a) *Ja*
   b) *Nein*

9) <u>Wie hoch war die höchste Dosierung von Pregabalin (Lyrica®) bei Ihren Patienten/Patientinnen?</u>
   a) *Unter 600mg/Tag*
   b) *Über 600 mg/Tag (Wie viel? ____________________)*

10) <u>Verordnen Sie selbst Pregabalin (Lyrica®) zur Behandlung von Entzugssymptomatik bei Abhängigkeit von Opioiden?</u>
   a) *Ja*
   b) *Nein*

11) Kennen Sie Entzugssymptomatiken bei Patienten/Patientinnen mit Pregabalin (Lyrica®)?
   a) *Ja*
   b) *Nein*

12) Falls ja, welche Entzugssymptomatiken sind Ihnen bekannt? *(Mehrfachantwort möglich)*
   a) *Palpitationen*
   b) *Schweissausbrüche*
   c) *Kältegefühle*
   d) *Zittern*
   e) *Hohe Aggressivität*
   f) *Selbstverletzungstendenz*
   g) *Depression*
   h) *Angetriebenheit*
   i) *Missmut*
   j) *Andere* ____________________

13) Haben Ihre Patienten/Patientinnen welche von Pregabalin (Lyrica®) abhängig sind als Entzugserskomplikation einen epileptischen Anfall erlebt?
   a) *Ja*
   b) *Nein*

14) Beobachten Sie gehäuft Verhaltensauffälligkeiten bei Patienten/Patientinnen unter Pregabalin (Lyrica®)?
   a) *Ja*
   b) *Nein*

15) Falls ja, welche Verhaltensauffälligkeiten beobachten Sie?
   a) *Fremdaggressivität*
   b) *Selbstaggressivität*
   c) *Andere* ____________________

16) Was machen Sie bei Patienten/Patientinnen die Verhaltensauffälligkeiten entwickeln?
   a) *Absetzen*
   b) *Reduzieren*
   c) *Weiterführen*
   d) *Substituieren durch andere Wirkstoffe (z.B. – Benzodiazepine, Valproinsäure…)*
      *Welche?*____________________

17) Welche Nationalität haben die Patienten/Patientinnen, die eine Abhängigkeit an Pregabalin (Lyrica®) haben?
   a) *Inländisch*
   b) *Ausländisch - Welche Nationalität?*____________________

18) Bringen Ihre Patienten/Patientinnen das Pregabalin (Lyrica®) mit aus ihren Heimatstaaten?
   a) *Ja*
   b) *Nein*

19) Wie lange nehmen Ihre Patienten/Patientinnen schon Pregabalin (Lyrica®) ein?
   a) *Weniger als 5 Jahren*
   b) *Mehr als 5 Jahren*

20) Wie wurde der Konsum vom Pregabalin (Lyrica®) Ihrer Patienten/Patientinnen initiiert?
   a) *Iatrogen*
   b) *Schwarzmarkt*

21) Was machen Sie bei Dosen über 600 mg (OFF Label)?
   a) *Absetzen*
   b) *Reduzieren*
   c) *Weiterführen*
   d) *Substituieren durch andere Wirkstoffe(Durch Z.b. – Benzodiazepines, Valproinsäure…) Welche?* ____________________

22) *Frage an Kolleginnen/Kollegen aus Deutschland:* Welche Maßregeln bzw. Sanktionen sind Ihre Patienten/Patientinnen unterworfen?
   a) §§ 63
   b) §§64 StGB

---

23) Wie alt sind Sie?

______________ Jahre alt

24) Was ist Ihr Geschlecht?
   a) *Weiblich*
   b) *Männlich*

25) In welchem Land sind sie als Arzt im Gefängnis/Forensik tätig?
   a) *Schweiz*
   b) *Deutschland*
   c) *Österreich*

26) In welcher Einrichtung sind Sie tätig?
   a) *Umfeld Medizin (Forensische Kliniken)*
   b) *Umfeld Justiz*

27) Falls Sie im Umfeld Justiz tätig sind, in welcher Einrichtung?
   a) *Gefängnis*
   b) *Massnahmezentrum*
   c) *Justizvollzuganstalten*

28) Haben Sie einen Facharzttitel?
   a) *Ja*
   b) *Nein*

29) Falls Sie einen Facharzttitel haben, welchen Schwerpunkt haben Sie?
   a) *Forensik*
   b) *Sucht*
   c) *Allgemeinmedizin*
   d) *Andere - Welche?* ________________________________

30) Seit wann sind Sie als Arzt tätig?

______
